# Epigenetic repression of phosphatidylethanolamine *N*-methyltransferase (*PEMT*) in *BRCA1*-mutated breast cancer

**DOI:** 10.18632/oncotarget.1800

**Published:** 2014-02-27

**Authors:** Da Li, Fang-Fang Bi, Na-Na Chen, Ji-Min Cao, Wu-Ping Sun, Yi-Ming Zhou, Chen Cao, Chun-Yan Li, Qing Yang

**Affiliations:** ^1^ Department of Obstetrics and Gynecology, Shengjing Hospital of China Medical University, Shenyang, China; ^2^ Department of Molecular Immunology, Graduate School of Medicine, Nagoya University, Nagoya, Japan; ^3^ Department of Physiology and Pathophysiology, Institute of Basic Medical Sciences, Chinese Academy of Medical Sciences, School of Basic Medicine Peking Union Medical College, Beijing, China; ^4^ Division of Cell Signaling, National Institute for Physiological Sciences, Okazaki, Japan; ^5^ Department of Pathology, Chinese PLA General Hospital, Beijing, China; ^6^ Department of Histology and Embryology, Institute of Basic Medical Sciences, Chinese Academy of Medical Sciences, School of Basic Medicine Peking Union Medical College, Beijing, China

**Keywords:** PEMT, DNA methylation, Histone modifications, BRCA1, Breast cancer

## Abstract

Phosphatidylethanolamine N-methyltransferase (*PEMT*) plays a critical role in breast cancer progression. However, the epigenetic mechanism regulating *PEMT* transcription remains largely unknown. Here, we show that the first promoter-specific transcript 1 is the major *PEMT* mRNA species, and methylation of the -132 site is a key regulatory element for the *PEMT* gene in *BRCA1*-mutated breast cancer. Mechanistically, hypermethylated -132 site-mediated loss of active histone marks H3K9ac and increase of repressive histone marks H3K9me enrichment synergistically inhibited *PEMT* transcription. Clinicopathological data indicated that a hypermethylated -132 site was associated with histological grade (*P* = 0.031) and estrogen receptor status (*P* = 0.004); univariate survival and multivariate analyses demonstrated that lymph node metastasis was an independent and reliable prognostic factor for *BRCA1*-mutated breast cancer patients. Our findings imply that genetic (e.g., *BRCA1* mutation) and epigenetic mechanisms (e.g., DNA methylation and histone modifications) are jointly involved in the malignant progression of *PEMT*-related breast cancer.

## INTRODUCTION

Breast cancer is the most common malignancy and a major cause of mortality in women worldwide [1]. Accumulating evidence indicates that an increased risk for breast cancer is associated with dietary factors [2] and a few significant-risk genetic components (e.g., *BRCA1*) [3]. *BRCA1* is a tumor suppressor gene which plays a key role in numerous cellular processes, including transcription regulation, DNA damage repair and protein ubiquitination.^4^ Recent research has confirmed that *BRCA1* is an important transcriptional regulator, and *BRCA1*-depleted breast cancer cells shows changes to approximately 7% of the mRNAs expressed [4]. Moreover, our recent study also indicated that angiotensin II type 1 receptor and epidermal growth factor receptor displayed different expression patterns in *BRCA1*-defective cancer cells [5,6], and confirmed that differential epigenetic regulation of transcription exist along with BRCA1 inactivation [7,8]. Therefore, one can speculate that there are wide ranges of gene expression and regulation differences between *BRCA1* dysfunction and the basal phenotype. To date, choline is among the well-studied essential nutrients that are involved in breast cancer; for example: (i) choline-containing compounds are significantly changed in breast cancer [9,10]; (ii) choline intake is inversely correlated with breast cancer risk [11-13]; and (iii) aberrant choline metabolism is often associated with malignant transformation, invasion, and metastasis of breast cancer [14-16]. Phosphatidylethanolamine *N*-methyltransferase (*PEMT*) is a small integral membrane protein that catalyzes the *de novo* synthesis of choline using *S*-adenosylmethionine as a methyl donor [17]. The human *PEMT* gene is located on chromosome 17p11.2 and consists of nine exons and eight introns, and differential promoter usage generates multiple transcripts [18]. It is interesting to note that functional polymorphisms of the *PEMT* gene play an important role in breast cancer development [12,19-21]. However, the expression patterns and transcriptional regulation of individual transcripts remain to be elucidated. Therefore, the present study was undertaken to investigate *PEMT* transcriptional regulation from genetic (*BRCA1* mutation or not) and epigenetic (promoter methylation and histone modifications) aspects, and to provide novel insight into epigenetic change-mediated abnormal *PEMT* expression in *BRCA1*-mutated breast cancer progression.

## RESULTS

### *BRCA1*-mutated breast cancer displayed a hypermethylated -132 site and promoter region

**Fig 1:**
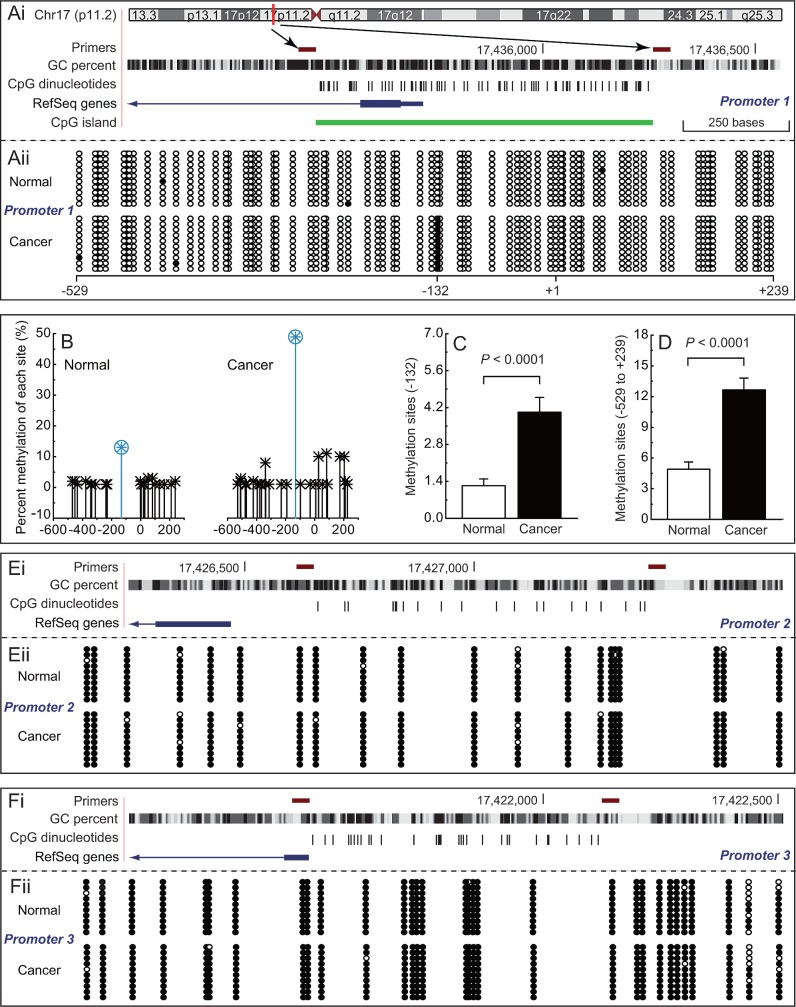
Methylation patterns of differential *PEMT* promoter in *BRCA1*-mutated breast cancer Ai, location of CpG sites in the first promoter of *PEMT*. Genomic coordinates are shown, along with the primer-amplified fragments, GC percentage, location of individual CpG dinucleotides (dashes), the *PEMT* RefSeq gene (exon 1 shown as a blue box and intron shown as an arrowed line), and CpG island (green bar). The arrow indicates the transcriptional direction, +1 is the transcription initiation site. Aii, comparative analysis of methylation patterns in the first promoter of *PEMT* in *BRCA1*-mutated breast cancer, and their adjacent normal breast tissues (each group, n = 68). The circles correspond to CpG sites denoted by the black dashes in Fig. 1Ai. Closed circles, methylation; open circles, unmethylation. Ten individual clones were sequenced for each sample. B, summary of the methylation patterns of the first *PEMT* promoter in Fig. 1Aii. The y-axis shows the mean methylation sites. C and D, overall methylation percentage of position -132 and the *PEMT* core promoter region (−529 to +239). Bar graphs show mean ± SD. E and F, comparative analysis of methylation patterns in the second and third promoters of *PEMT*, respectively (each group, n = 68)

To investigate the epigenetic regulation of *PEMT*, we compared the DNA methylation patterns of three promoters between *BRCA1*-mutated or non-mutated breast cancer and their adjacent normal breast tissues. As shown in Fig. 1Aii and B, *BRCA1*-mutated breast cancer exhibited hypermethylation of the first promoter (*P* < 0.001; Fig. 1D), especially around the -132 site (*P* < 0.001; Fig. 1C). However, no significant methylation differences were observed in the second and third promoters in *BRCA1*-mutated breast cancer (Figs. 1E and F), and in all three promoters in non-mutated breast cancer (Supplementary Fig. S1A), compared with normal breast tissues.

### Low *PEMT* transcript 1 levels showed a significant inverse correlation with hypermethylation of the -132 site in *BRCA1*-mutated breast cancer

**Fig 2:**
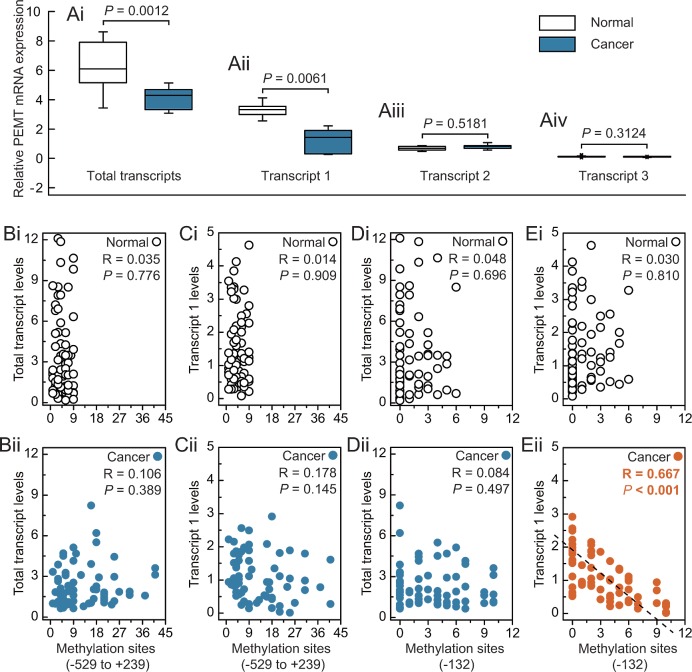
Expression features of *PEMT* in *BRCA1*-mutated breast cancer A, relative *PEMT* mRNA levels of differential promoter utilization (each group, n = 68). B and C, correlation between the methylated sites (−592 to +239), and total transcript or transcript 1 levels in *BRCA1*-mutated breast cancer and adjacent normal breast tissues, respectively. D and E, correlation between the methylated sites (-132), and total transcript or transcript 1 levels in *BRCA1*-mutated breast cancer and adjacent normal breast tissues, respectively. Open circles, normal breast tissues. Closed circles, breast cancer tissues (each group, n = 68).

Multiple promoters of the human *PEMT* gene generate at least three transcripts and exhibit tissue-specific expression; for example, transcript 1, transcript 2, and transcript 3 are mainly initiated from the first, second, and third promoter, respectively. Our results indicated that transcript 1 was the major *PEMT* mRNA species in human breast tissues, and reduced *PEMT* mRNA may be dependent on reduced levels of the first-promoter-specific transcript 1 in human breast tissues (Fig. 2A and Supplementary Fig. S1B). In addition, expression levels of *PEMT* were decreased in *BRCA1*-mutated breast cancer compared to their adjacent normal breast tissues. However, no expression differences for different transcripts between non-*BRCA1*-mutated breast cancer and their adjacent normal breast tissues were observed (Supplementary Fig. S1B). In addition, we analyzed the correlation between total transcript levels or transcript 1 levels, and the number of methylated sites at -132 site or around the *PEMT* core promoter region (−529 to +239) in *BRCA1*-mutated breast cancer and normal breast tissues (Figs. 2B–E). It is interesting to note that a significant inverse correlation was only observed between levels of *PEMT* transcript 1 and numbers of methylated -132 sites (*R* = 0.667, *P* < 0.001; Fig. 2Eii).

### Hypermethylated -132 site is a key regulatory mechanism for *PEMT* transcription in *BRCA1*-mutated breast cancer

**Fig 3:**
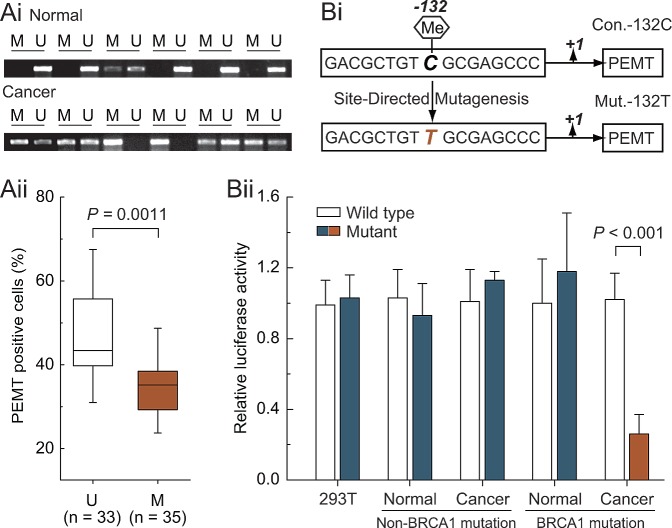
Methylated -132 site and *PEMT* transcriptional activity Ai, comparative analysis of -132 site methylation between *BRCA1*-mutated breast cancer and normal breast tissues using methylation-specific PCR. Aii, relationship between *PEMT* protein expression and promoter methylation in *BRCA1*-mutated breast cancer (U, unmethylated group, n = 33; M, methylated group, n = 35). Bi, the schematic represents the nucleotide sequence around the -132 site (Con.-132C) that was point mutated at position -132 (C to T) to generate the Mut.-132T. Bii, 293T cells (repeated 12 times), and primary non-mutated (n = 75) and *BRCA1*-mutated breast cancer (n = 68) and their normal breast cells were transfected with Con.-132C and Mut.-132T. At 24 hours after transfection, whole-cell extracts were analyzed for luciferase activity. Bar graphs show mean ± SD.

Based on the results above, we further explored the relationship between -132 site methylation and *PEMT* expression in *BRCA1*-mutated breast cancer specimens. *BRCA1*-mutated cancer specimens were classified into unmethylated and methylated groups for comparing their protein expression of *PEMT*. The methylated group showed a significantly lower expression of *PEMT* compared with the unmethylated group (*P* = 0.0011; Fig. 3Ai and Aii). To further confirm the role of the cytosine located at the -132 site, a point mutation of cytosine to thymine was constructed (Fig. 3Bi). Notably, -132 was the critical site for *PEMT* transcription only in primary *BRCA1*-mutated breast cancer cells (Fig. 3Bii). Moreover, methylated -132 site can also significantly inhibit the transcription of *PEMT* (Supplementary Fig. S2A and B).

### Loss of H3K9ac and increase of H3K9me enrichment around the methylated -132 site in *BRCA1*-mutated breast cancer

**Fig 4:**
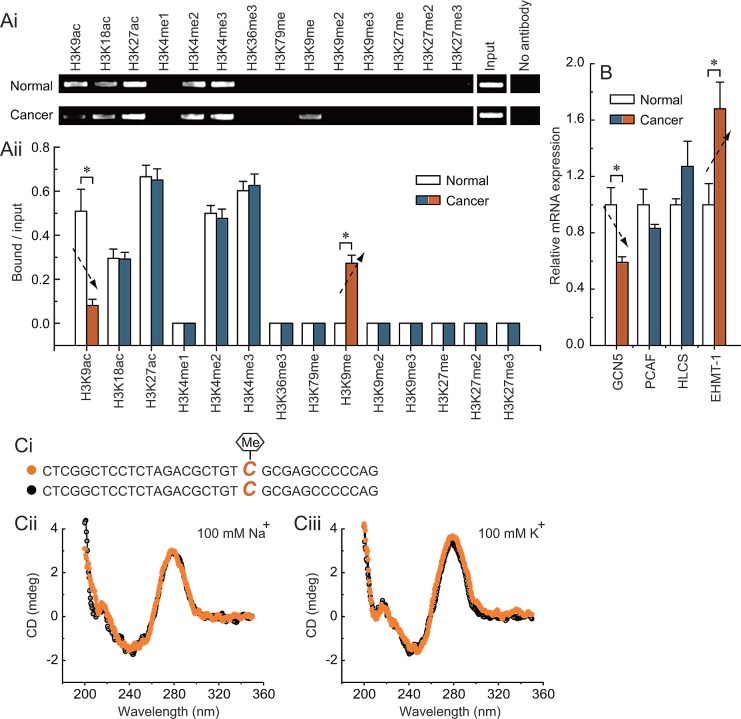
Characteristic histone modification pattern of -132 site methylation in *BRCA1*-mutated breast cancer Ai, chromatin immunoprecipitation was performed using antibodies to H3K9ac, H3K18ac, H3K27ac, H3K4me1, H3K4me2, H3K4me3, H3K36me3, H3K79me, H3K9me, H3K9me2, H3K9me3, H3K27me, H3K27me2, and H3K27me3. PCR was performed for regions around the -132 site. A negative control without antibodies is included for comparison. Aii, representative results of primary *BRCA1*-mutated breast cancer and their normal breast tissues are shown (each group, n = 68). Bar graphs show mean ± SD. * *P* < 0.05 *vs*. normal. B, expression levels of the chromatin-modifying enzymes *GCN5*, *PCAF*, *HLCS*, and *EHMT-1* in *BRCA1*-mutated breast cancer and normal breast tissues (each group, n = 68). Bar graphs show mean ± SD. * *P* < 0.05 *vs*. normal. Ci, the schematic represents the selected nucleotide sequence with or without a methyl group at the fifth position of the cytosine pyrimidine ring at position -132. Cii and Ciii, the CD spectra of the selected nucleotide sequence in the presence of 100 mM Na^+^ or 100 mM K^+^ are shown

To obtain further understanding of the regulatory potential of the crosstalk between DNA methylation and histone modification in controlling *PEMT* transcription, we examined the active histone markers H3K9ac, H3K18ac, H3K27ac, H3K4me1, H3K4me2, H3K4me3, H3K36me3, and H3K79me, and the repressive histone markers H3K9me, H3K9me2, H3K9me3, H3K27me, H3K27me2, and H3K27me3 in the first promoter of *PEMT*, especially around the -132 site. Chromatin immunoprecipitation analysis indicated that the levels of H3K9ac were reduced, and levels of H3K9me were increased around the methylated -132 site in *BRCA1*-mutated breast cancer (Fig. 4A). Chromatin-modifying enzymes that facilitate the creation of H3K9ac and H3K9me were also analyzed: *GCN5* and *PCAF* are capable of creating H3K9ac; *HLCS* and *EHMT-1* are capable of creating H3K9me. Although there was no significant change in the expression of *PCAF* and *HLCS*, the expression level of *GCN5* was reduced, and *EHMT-1* was increased in *BRCA1*-mutated breast cancer (Fig. 4B; *P* < 0.05). In addition, there was little difference in histone markers around the -132 site between *BRCA1* wild type and mutant tissue, such as the lack of specific H3K9me in *BRCA1* wild type tissue. However, no differences were found for histone markers around the -132 site between normal and cancer tissues in *BRCA1* wild type cases (Supplementary Fig. S3).These results, together with the methylation data in Figs. 1, 2, and 3, suggest that *PEMT* transcription may be associated with changes in epigenetic features, including reduced *GCN5*-related H3K9ac enrichment and increased *EHMT-1*-related H3K9me enrichment around the hypermethylated -132 site in *BRCA1*-mutated breast cancer. Recently, a substantial body of evidence suggests that most of the known genes contain specific motifs in their promoter regions, which can modulate gene transcription by affecting the binding of histone [22]. Therefore, CD spectra were used to gain information on whether methylation of the -132 site associated abnormal H3K9ac and H3K9me enrichment, which may involve changes in *PEMT* promoter structure. However, our results showed that -132 site methylation may not affect the structure of the first promoter of *PEMT* (Fig. 4C).

### H3K9ac and H3K9me present at the -132 site are responsible for transcriptional regulation of *PEMT* in *BRCA1*-mutated breast cancer

**Fig 5:**
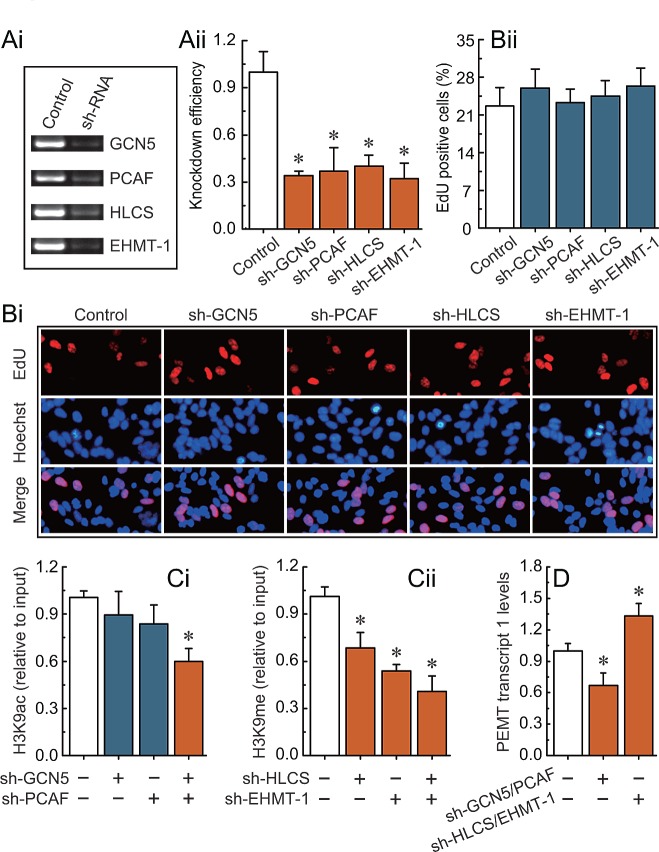
H3K9ac and H3K9me-mediated transcriptional regulation of *PEMT* in *BRCA1*-mutated breast cancer cells Ai, RT-PCR showing *GCN5*, *PCAF*, *HLCS*, and *EHMT-1* levels before and after knockdown by shRNAs, and normalized to β-actin expression. Aii, the results from three independent experiments are represented as mean ± SD. Bi, EdU labeling showing the proliferation of *GCN5*-, *PCAF*-, *HLCS*-, and *EHMT-1*-silenced in primary *BRCA1*-mutated breast cancer cells (each group, n = 68). Blue, Hoechst 33342 labeling of cell nuclei; red, EdU labeling of nuclei of proliferative cells. Bii, the EdU incorporation rate was expressed as the ratio of EdU-positive cells to total Hoechst 33342-positive cells. Ci, analysis of histone modification H3K9ac enrichment around the -132 site after the deletion of *GCN5* and *PCAF* in primary *BRCA1*-mutated breast cancer cells (each group, n = 68). Cii, analysis of histone modification H3K9me enrichment around the -132 site after the deletion of *HLCS* and *EHMT-1* in primary *BRCA1*-mutated breast cancer cells (each group, n = 68). D, *PEMT* transcript 1 levels after the deletion of H3K9ac and H3K9me around the -132 site in primary *BRCA1*-mutated breast cancer cells (each group, n = 68). Bar graphs show mean ± SD. * *P* < 0.05 *vs*. control.

We observed that the knockdown of *GCN5*, *PCAF*, *HLCS*, and *EHMT-1* (Fig. 5A) had no detectable effect on cell morphology and proliferation (Fig. 5B). Combined knockdown of *GCN5* and *PCAF*, or *HLCS* and *EHMT-1* specifically induced a decrease in H3K9ac or H3K9me enrichment around the -132 site, respectively (Figs. 5Ci and Cii). Notably, after the deletion of H3K9ac or H3K9me, the transcription of *PEMT* was significantly down-regulated or up-regulated, respectively (Fig. 5D). The phenomenon was further confirmed by other specific siRNAs (Supplementary Fig. S4).

### Correlation of -132 site methylation with clinicopathological characteristics in *BRCA1*-mutated breast cancer

The correlation between -132 site methylation and clinicopathological parameters was analyzed using Fisher's exact test. As shown in Table 1, *PEMT* methylation was associated with histological grade (*P* = 0.031) and estrogen receptor status (*P* = 0.004). No significant associations were observed between *PEMT* methylation and age at diagnosis, menstrual status, tumor size, lymph node, progesterone receptor, c-erbB-2, p53, Ki67, or E-cadherin status.

**Table 1:** Association between *PEMT* promoter methylation and clinicopathological features of *BRCA1*-mutated breast cancer

	n	M	(%)	UM	(%)	P
Age at diagnosis						0.799
≤ 50 y	23	11	31.43	12	36.36	
> 50 y	45	24	68.57	21	63.64	
Menstrual status						1.000
Premenopausal	28	14	40.00	14	42.42	
Postmenopausal	40	21	60.00	19	57.58	
Tumor size						0.137
≤ 5 cm	43	19	54.29	24	72.73	
> 5 cm	25	16	45.71	9	27.27	
Histological grade						0.031
I-II	49	21	60.00	28	84.85	
III	19	14	40.00	5	15.15	
LN status						0.809
Positive	28	15	42.86	13	39.39	
Negative	40	20	57.14	20	60.61	
ER status						0.004
Positive	37	13	37.14	24	72.73	
Negative	31	22	62.86	9	27.27	
PR status						0.144
Positive	41	18	51.43	23	69.70	
Negative	27	17	48.57	10	30.30	
c-erbB-2 status						1.000
Positive	29	15	42.86	14	42.42	
Negative	39	20	57.14	19	57.58	
p53 status						0.300
Positive	21	13	37.14	8	24.24	
Negative	47	22	62.86	25	75.76	
Ki67 status						0.406
Positive	51	28	80.00	23	69.70	
Negative	17	7	20.00	10	30.30	
E-cadherin status						0.767
Positive	54	27	77.14	27	81.82	
Negative	14	8	22.86	6	18.18	

M, methylated; UM, unmethylated; LN, lymph node; ER, estrogen receptor; PR, progesterone receptor.

### Multivariate and univariate analysis of overall survival for patients with *BRCA1*-mutated breast cancer

We analyzed overall survival to assess the prognostic significance of clinicopathological parameters. Multivariate Cox regression analysis indicated that lymph node metastasis (*P* = 0.090), estrogen receptor (*P* = 0.149), progesterone receptor (*P* = 0.157), and p53 status (*P* = 0.136) showed a trend for an independent prognostic factor for predicting the overall survival of *BRCA1*-mutated breast cancer patients (Supplementary Table S1). We also performed the Kaplan-Meier analysis and log-rank tests for overall survival in defining prognostic subgroups. The results revealed that lymph node metastasis (Supplementary Fig. S5E, *P* < 0.05) and E-cadherin status (Supplementary Fig. S5K, *P* < 0.05) were significant prognostic factors. Moreover, patients with high histological grade (Supplementary Fig. S5D, *P* = 0.248) or estrogen receptor-negative (Supplementary Fig. S5F, *P* = 0.278), c-erbB-2-positive (Supplementary Fig. S5H, *P* = 0.244), p53-negative (Supplementary Fig. S5I, *P* = 0.072), or Ki67-positive (Supplementary Fig. S5J, *P* = 0.145) breast cancer showed a trend for poor overall survival, although not statistically significant. No significant difference in overall survival was found among patients with different age at diagnosis, premenopausal, tumor size, progesterone receptor status, or *PEMT* methylation (Supplementary Fig. S5A, B, C, G, and L).

## DISCUSSION

Promoter methylation, with concurrent changes in histone modifications, is an epigenetic phenomenon that can affect the conformation of chromatin and tissue-specific gene expression[23,24]. In this study, we report that the first-promoter-specific transcript 1 is the major *PEMT* mRNA species in human breast tissues, and that methylation of the -132 site is a key regulatory element for *PEMT* transcription in *BRCA1*-mutated breast cancer. The molecular mechanism may involve the hypermethylated -132 site-mediated loss of active histone marker H3K9ac and an increase of the repressive histone marker H3K9me enrichment, which synergistically inhibit the transcription of *PEMT*. Interestingly, the synergistic inhibitory effect of hypermethylated -132 site and H3K9ac and H3K9me histone modification were only observed primarily in cells originating from *BRCA1*-mutated breast cancer; the transformed cell line 293T and non-mutated breast cancer were insensitive to the loss of H3K9ac and H3K9me enrichment around the -132 site in the first promoter of *PEMT*. Accordingly, specific regulatory mechanisms may exist, and *PEMT* expression is likely to be the result of a complex interaction of multiple factors in *BRCA1*-mutated breast cancer cells, such as changes in chromatin-modifying enzymes that reduce *GCN5* levels and increase *EHMT-1* levels. Remarkably, as shown in Supplementary Fig. S6, *BRCA1*-mutated breast cancer tissues exhibited lower levels of choline compared with normal breast tissues and non-mutated cancer tissues. Choline is known to be an effective methyl donor that is involved in DNA methylation. This may help to explain why global DNA methylation was further reduced in *BRCA1*-mutated breast cancer (Supplementary Fig. S7). Moreover, DNA methyltransferase 1 *(DNMT1)* plays an important role in maintaining DNA methylation patterns [25,26], Shukla showed that *DNMT1* was a transcriptional target of *BRCA1* [27]. Our recent study confirmed that *DNMT1* mRNA and protein were decreased in *BRCA1*-mutated breast cancer.^7^ Therefore, abnormal *BRCA1* gene-mediated reduced levels of *DNMT1* may also be involved in the progression of genomic DNA hypomethylation in breast cancer. In addition, Xu observed that low choline levels were associated with more frequent *BRCA1* promoter methylation-mediated expression inhibition [28]. It can be speculated that dynamic crosstalk may exist between *PEMT*-related choline synthesis and choline-related *BRCA1* inactivity. Meanwhile, choline plays a critical role in the methionine cycle [29]. Beetstra confirmed that defects in methionine metabolism may be related to breast cancer risk in BRCA carriers [30]. These results, together with our observations, suggest that low levels of *PEMT* may be involved in the development of *BRCA*-mutated breast cancer through choline deficiency.

Promoter hypermethylation is often associated with adverse clinical events [31]. In line with this, clinicopathological data indicated that hypermethylation of the -132 site may be an effective indicator for histological grade and estrogen receptor status in *BRCA1*-mutated breast cancer tissues (Table 1). Moreover, univariate survival and multivariate analyses indicated that lymph node metastasis was an independent and reliable prognostic factor which is associated with worse outcomes for *BRCA1*-mutated breast cancer patients.

This study provides new insights into the causes and prognosis of *PEMT* inactivation in *BRCA1*-mutated breast cancer. The mechanism involves the synergistic effects of promoter methylation and histone modification. Therefore, a more specific epigenetic therapy could be developed for *BRCA1*-mutated breast cancer.

## METHODS

### Ethics Statement

Investigation has been conducted in accordance with the ethical standards and according to the Declaration of Helsinki and according to national and international guidelines and has been approved by the authors' institutional review board.

### Patients and tissue collection

Sixty-eight invasive ductal carcinomas from *BRCA1* mutation carriers and 75 invasive ductal carcinomas from non-*BRCA1* mutation carriers were enrolled between 2007 and 2009, and all patients gave informed consent. Fresh breast cancer and adjacent normal breast tissues were obtained at the time of primary surgery before any chemotherapy or radiotherapy. Hematoxylin and eosin staining of the samples for histopathological diagnosis and grading were performed by three staff pathologists using the Nottingham Combined Histologic Grade. The tumor stages were classified according to the National Comprehensive Cancer Network guidelines. Their characteristics are given in Supplementary Table S2.

### Cell culture, lentiviral infection, and cell proliferation assay

Detailed isolation and cultivation protocols were established as previously described [32]. Briefly, tissues were washed, minced, and digested in 0.1% collagenase type III (Sigma, CA, USA) overnight at 37°C. The suspension was filtered through a 100-μm nylon mesh to remove the remaining clumps. Following gentle centrifugation at 100 g for 5 min, the epithelial and stromal fractions were cultured in Dulbecco's Modified Eagle Medium (DMEM) with 10% fetal bovine serum (Invitrogen, CA USA) for 24 h to promote cell attachment. Breast epithelial cells were maintained in CnT-27 mammary epithelium medium (CELLnTEC, Bern, Switzerland), and used in all experiments were passage 2 to passage 5. Human 293T cells were maintained in DMEM with 10% fetal bovine serum (Invitrogen), and have been tested and authenticated at Jun 1th, 2012. The short hairpin RNAs (shRNAs) lentiviral particles of histone acetyltransferase (*GCN5*), p300/ CBP-associated factor (*PCAF*), Holocarboxylase synthetase (*HLCS*) and Euchromatic histone-lysine *N*-methyltransferase 1 (*EHMT-1*) were purchased from Santa Cruz Biotechnology (CA, USA), see details in Supplementary Table S3. Transfections were performed using polybrene and enhanced infection solution (Genechem, Shanghai, China) according to the manufacturer's recommended protocol. The knowdown effiency was confirmed by RT-PCR and western blotting (Supplementary Fig. S8). After 48-hour infection, cell proliferation was determined using the Cell-Light™ EdU Apollo^®^643 In Vitro Imaging Kit (Ribobio, Guangzhou, China) following the manufacturer's instructions.

### DNA methylation analysis

Genomic DNA from the breast cancer and adjacent normal breast tissues was extracted using the TIANamp Genomic DNA kit (Tiangen Biotech, Beijing, China). Sodium bisulfite conversion, PCR amplification, and general experimental procedures are described in the Supplementary Methods. The specific primer sequences for bisulfite sequencing and methylation-specific PCR are shown in Supplementary Table S4. Genomic DNA methylation assay are described in the Supplementary Methods.

### Real-time PCR, immunohistochemistry and western blotting analysis

Real-time PCR, immunohistochemistry and western blotting analysis are described in the Supplementary Methods. The specific primer sequences for real-time PCR are shown in Supplementary Table S4. The primary antibody for immunohistochemistry and western blotting analysis are shown in Supplementary Table S3.

### Chromatin immunoprecipitation (ChIP), site-directed mutagenesis, transfection, and dual-luciferase reporter assay

ChIP, site-directed mutagenesis, transfection, and dual-luciferase reporter assay are described in the Supplementary Methods. The specific primer sequences for site-directed mutagenesis and ChIP are shown in Supplementary Table S4. The specific antibodies for ChIP are shown in Supplementary Table S3.

### Circular dichroism (CD) spectra

CD spectra were obtained using a Jasco J-810 spectropolarimeter at 25°C using a 0.1 cm path length cell. Data were collected with a 2 nm slit width from 350 to 200 nm at 0.5 nm intervals and averaged over three scans. CD experiments were carried out on DNA samples (5 μM) using a buffer containing 0.2 M phosphate buffer (pH 7.0) in the presence of 100 mM Na^+^ or K^+^. The DNA samples were annealed by heating to 95°C for 5 min followed by cooling to room temperature over 10 h before analysis. The DNA sequence was as follows: 5-CTCGGCTCCTCTAGACGCTGT “C (CH3 or non-CH3)” GCGAGCCCCCAG, and synthesized by Sangon Biotech Ltd (Shanghai, China).

### Measurement of choline levels

Tissue choline content was measured using the Choline/Acetylcholine Quantification kit (Biovision, Mountain View, CA, USA) following the manufacturer's instructions. The absorbance of each plate was measured at 570 nm using a Bio-Rad model 550 microplate reader (Hercules, CA, USA).

### Statistical analysis

Regression analysis was used to examine the possible relationship between *PEMT* mRNA and status of promoter methylation. The association between clinicopathological features and *PEMT* promoter methylation was determined using Fisher's exact test. Univariate analysis of survival was performed using the Kaplan-Meier method. Multivariate Cox regression analysis was performed to identify independent prognostic factors for overall survival. The data are presented as mean ± standard deviation (SD). Statistical differences in the data were evaluated by Student's t test or one-way analysis of variance (ANOVA) as appropriate, and were considered significant at *P* < 0.05.

## SUPPLEMENTARY FIGURES TABLES AND METHODS






