# Supplementary material for: Epigenetic repression of phosphatidylethanolamine N-methyltransferase (PEMT) in BRCA1-mutated breast cancer
Source: Oncotarget. 2014 Feb 27;5(5):1315–25. doi: 10.18632/oncotarget.1800 (PMC4012741; doi:10.18632/oncotarget.1800)
Supplement: Supplementary file 1 [file oncotarget-05-1315-s001.pdf]

Epigenetic repression of phosphatidylethanolamine *N*-methyltransferase (*PEMT*) in *BRCA1*-mutated breast cancer – Li et al

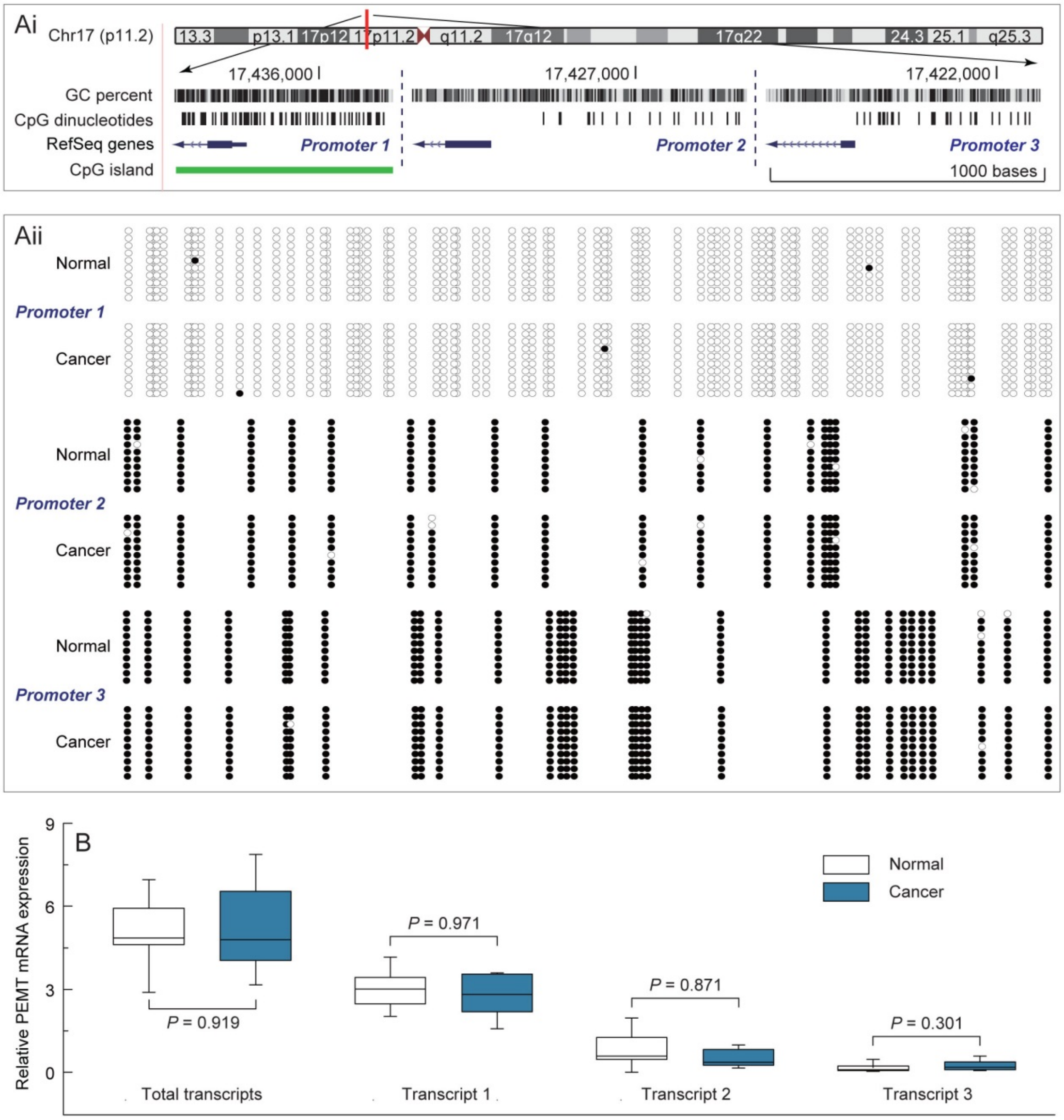

Fig. S1. Methylation and expression patterns of differential *PEMT* promoter in non-*BRCA1*-mutated breast cancer. Ai, location of CpG sites in the three promoters of *PEMT*. Genomic coordinates are shown, along with the primer-amplified fragments, GC percentage, location of individual CpG dinucleotides (dashes), the *PEMT* RefSeq gene (exon 1 shown as a blue box and intron shown as an arrowed line), and CpG island (green bar). The arrow indicates the transcriptional direction. Aii, comparative analysis of methylation patterns in the three promoter of *PEMT* in non-*BRCA1*-mutated breast cancer, and their adjacent normal breast tissues. The circles correspond to CpG sites denoted by the black dashes in Supplementary Fig. S1Ai. Closed circles, methylation; open circles, unmethylation. Ten individual clones were sequenced for each sample. B, relative *PEMT* mRNA levels of differential promoter utilization.

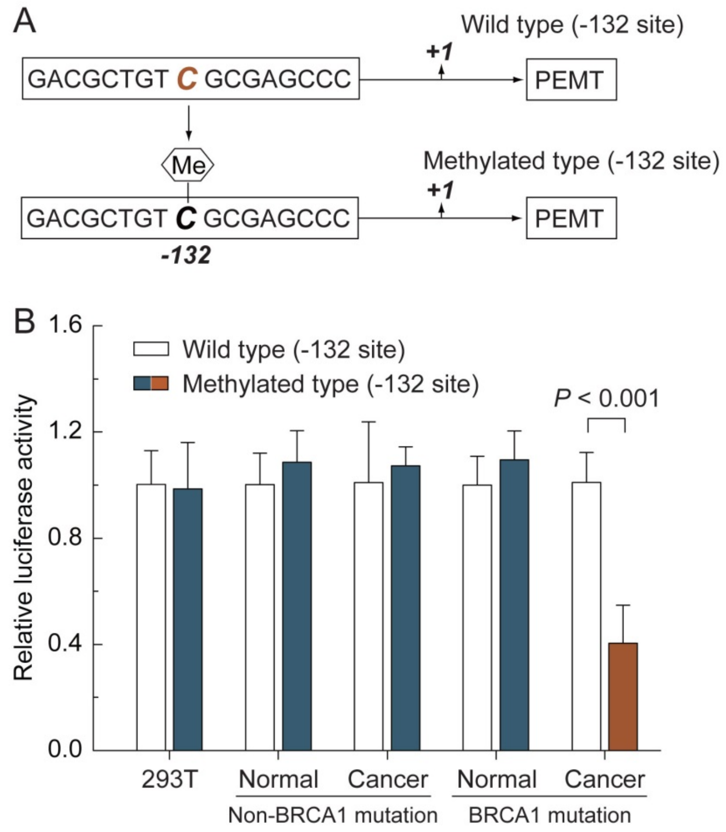

Fig. S2. Methylated -132 site-mediated *PEMT* transcriptional inhibition in *BRCA1*-mutated breast cancer cells. A, the schematic represents the nucleotide sequence around the -132 site (wild type) that was methylated at position -132 to generate the methylated type. B, 293T cells (repeated 12 times), and primary non-mutated (n = 75) and *BRCA1*-mutated breast cancer (n = 68) and their normal breast cells were transfected with wild type and methylated type plasmid. At 24 hours after transfection, whole-cell extracts were analyzed for luciferase activity. Bar graphs show mean ± SD.

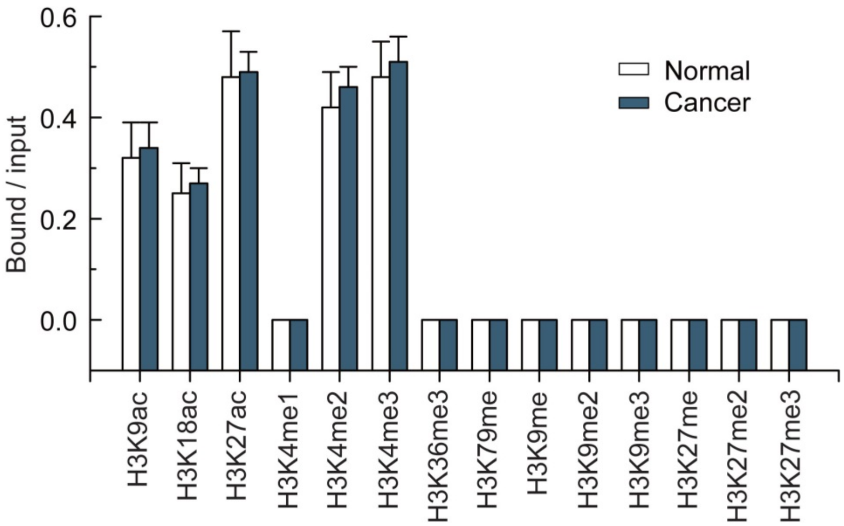

Fig. S3. Characteristic histone modification pattern of -132 site methylation in non-*BRCA1*-mutated breast cancer. A, chromatin immunoprecipitation was performed using antibodies to H3K9ac, H3K18ac, H3K27ac, H3K4me1, H3K4me2, H3K4me3, H3K36me3, H3K79me, H3K9me, H3K9me2, H3K9me3, H3K27me, H3K27me2, and H3K27me3. Representative results of 75 primary *BRCA1*-mutated breast cancer and their normal breast tissues are shown. Bar graphs show mean ± SD.

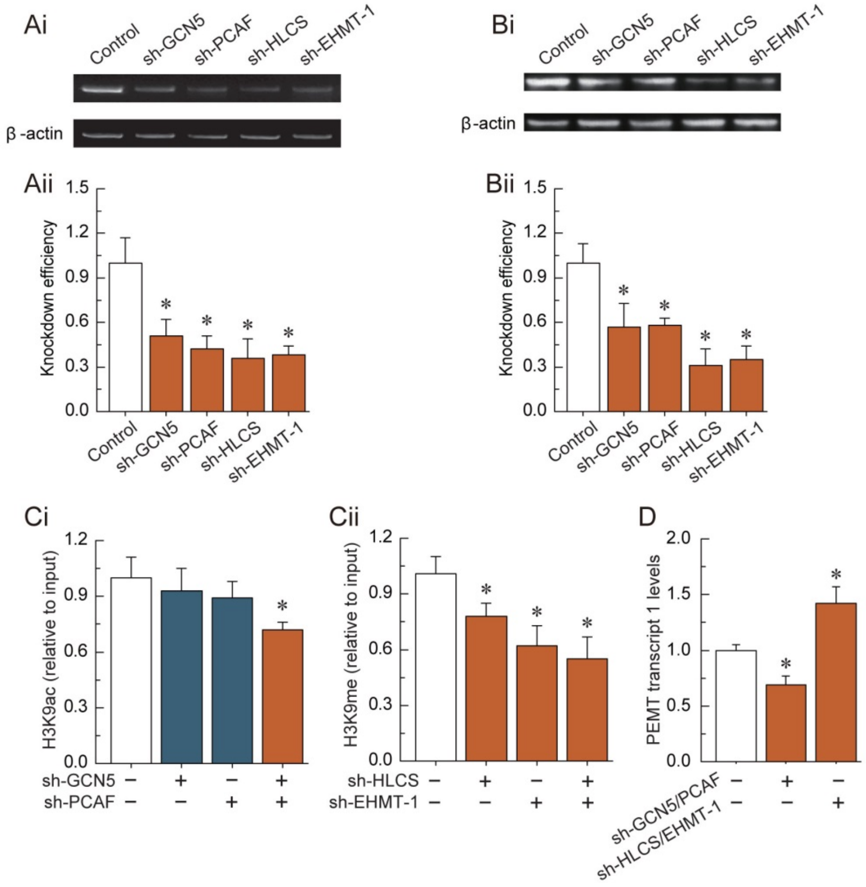

Fig. S4. H3K9ac and H3K9me-mediated transcriptional regulation of *PEMT* in *BRCA1*-mutated breast cancer cells. Ai and Bi, RT-PCR and western blot analysis showing GCN5, PCAF, HLCS, and EHMT-1 levels before and after knockdown by shRNAs, and normalized to □-actin expression. Aii and Bii, the results from three independent experiments are represented as mean ± SD. * $P < 0.05$ *vs*. control. Ci, analysis of histone modification H3K9ac enrichment around the -132 site after the deletion of GCN5 and PCAF by the second specific shRNAs in primary *BRCA1*-mutated breast cancer cells. Cii, analysis of histone modification H3K9me enrichment around the -132 site after the deletion of HLCS and EHMT-1 by the second specific shRNAs in primary *BRCA1*-mutated breast cancer cells. Bar graphs show mean ± SD. * $P < 0.05$ *vs*. control. D, *PEMT* transcript 1 levels after the deletion of H3K9ac and H3K9me around the -132 site in primary *BRCA1*-mutated breast cancer cells (each group, n = 68). Bar graphs show mean ± SD. * $P < 0.05$ *vs*. control.

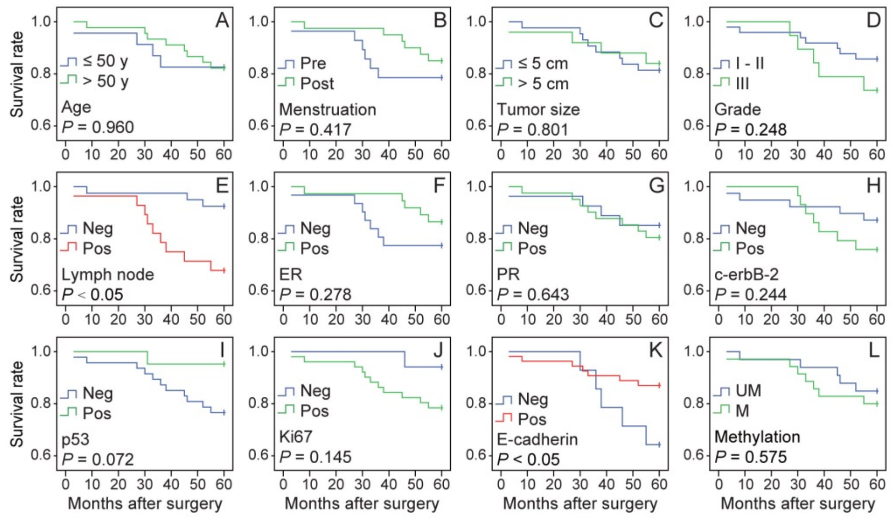

Fig. S5. Kaplan-Meier analysis of overall survival for 68 *BRCA1*-mutated breast cancer patients. The following variables were analyzed: age at diagnosis, menstruation, tumor size, grade, lymph node, estrogen receptor, progesterone receptor, c-erbB-2, p53, Ki67, E-cadherin, and *PEMT* methylation. Pre, premenopausal; Post, postmenopausal; ER, estrogen receptor; PR, progesterone receptor; Neg, negative; Pos, positive; UM, unmethylated; M, methylated.

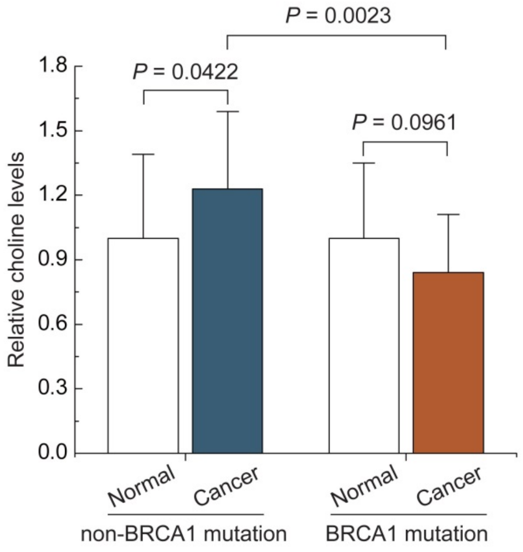

Fig. S6. Compared of tissues choline levels between *BRCA1*-mutated or non-mutated breast cancer and normal breast tissues.

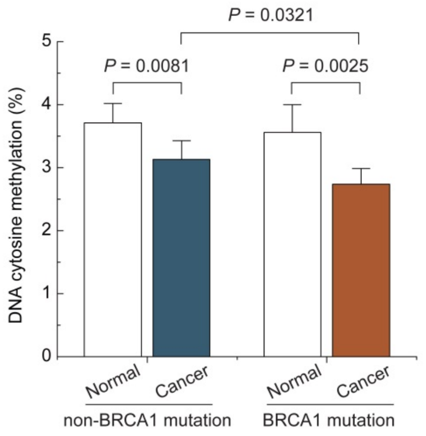

Fig. S7. Compared of global DNA methylation levels between *BRCA1*-mutated or non-mutated breast cancer and normal breast tissues.

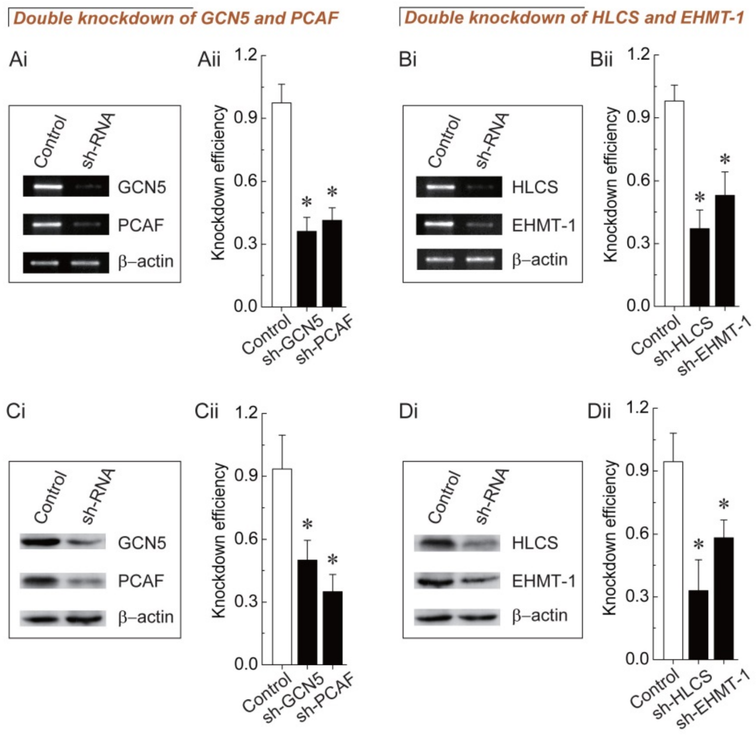

Fig. S8. Knowdown effiency in *BRCA1*-mutated breast cancer cells. Ai and Bi, RT-PCR showing GCN5 and PCAF, and HLCS and EHMT-1 levels before and after knockdown by shRNAs, and normalized to □-actin expression. Aii and Bii, the results from three independent experiments are represented as mean ± SD. * $P < 0.05$ *vs*. control. C and D, the RT-PCR results were confirmed by western blotting, and the results from three independent experiments are represented as mean ± SD. * $P < 0.05$ *vs*. control.
