# Supplementary material for: Epigenetic repression of phosphatidylethanolamine N-methyltransferase (PEMT) in BRCA1-mutated breast cancer
Source: Oncotarget. 2014 Feb 27;5(5):1315–25. doi: 10.18632/oncotarget.1800 (PMC4012741; doi:10.18632/oncotarget.1800)
Supplement: Supplementary file 2 [file oncotarget-05-1315-s002.pdf]

Supplementary Table 1. Prognostic factors for overall survival by multivariate Cox regression analysis

Variable	RR	95% CI	*P*
**Age at diagnosis** (> 50 y *vs* ≤ 50 y)	1.090	0.992-1.198	0.074
**Menstrual status (**Post *vs* Pre**)**	0.312	0.032-3.005	0.314
**Tumor size** (> 5 cm *vs* ≤ 5 cm)	0.911	0.177-4.677	0.911
**Histological grade** (III vs I-II)	0.939	0.117-7.523	0.953
**LN status (**Pos *vs* Neg**)**	4.533	0.790-26.002	0.090
**ER status (**Pos *vs* Neg**)**	0.144	0.010-2.003	0.149
**PR status (**Pos *vs* Neg**)**	3.784	0.600-23.853	0.157
**c-erbB-2 status (**Pos *vs* Neg**)**	2.054	0.381-11.076	0.403
**p53 status (**Pos *vs* Neg**)**	0.182	0.019-1.711	0.136
**Ki67 status (**Pos *vs* Neg**)**	2.308	0.190-28.048	0.511
**E-cadherin status (**Pos *vs* Neg**)**	0.486	0.066-3.588	0.479
**Methylation** (M *vs* UM)	0.422	0.057-3.099	0.396

List of abbreviations used: Post, postmenopausal; Pre, premenopausal; LN, lymph node; ER, estrogen receptor; PR, progesterone receptor; Pos, positive; Neg, negative; M, methylated; UM, unmethylated; RR, relative risk; CI, confidence interval.

Supplementary Table 2. Clinical characteristics for the 68 BRCA1-mutated breast cancer patients

Case	Age	Grade	LN	ER	PR	c-erbB-2	p53	Ki67	E-cadherin	Exon	Mutation	AA change	Mutation type[a]
1	49	I	+	-	+	+	-	+	+	2	c.65 T>A	p.L22X	NS
2	35	III	+	-	+	-	-	+	+	2	c.72 T>A	p.C24X	NS
3	41	II	+	-	-	-	-	+	+	3	c.120 C>G	p.D40E	MS
4	85	III	+	+	+	-	-	+	-	5	c.168 A>T	p.K56N	MS
5	62	III	+	-	-	+	-	+	-	5	c.199 G>C	p.D67H	MS
6	47	III	+	-	+	+	-	+	-	6	c.213 G>C	p.R71S	MS
7	52	III	+	-	+	+	-	+	-	5	c.197-198delAT	p.N66RfsX14	FS
8	75	I	-	-	-	-	-	+	+	6	c.243 A>T	p.Q81H	MS
9	60	III	-	-	-	-	-	+	+	6	c.273 T>A	p.C91X	NS
10	60	I	+	-	-	+	+	+	-	7	c.317 A>T	p.N106I	MS
11	36	II	-	+	+	-	+	+	+	7	c.349 C>G	p.H117D	MS
12	45	III	+	-	-	-	-	+	+	7	c.390 C>G	p.Y130X	NS
13	70	III	-	-	-	+	+	+	+	7	c.390delC	p.Y130X	NS
14	53	I	+	-	-	+	-	+	+	8	c.470-471delCT	p.S157X	NS
15	45	III	+	-	-	+	+	+	+	8	c.512 T>C	p.I171T	MS
16	56	II	+	+	-	-	+	+	+	10	c.635 C>G	p.T212S	MS
17	69	II	-	+	+	-	+	-	+	11	c.1117 del A	p.I373X	NS
18	60	III	-	+	+	-	-	-	+	11	c.2566 T>C	p.Y856H	MS

19	43	II	+	-	+	+	-	-	+	11	c.1245-1246delTC	p.L416KfsX3	FS
20	56	III	-	+	+	-	-	+	+	11	c.2363 T>C	p.V788A	MS
21	67	I	-	-	-	+	+	+	-	11	c.1475 T>C	p.I492T	MS
22	41	II	-	+	+	-	+	-	-	11	c.2429 A>G	p.N810S	MS
23	58	I	-	-	-	-	-	+	+	11	c.1808-1809delCA	p.S603X	NS
24	57	II	-	-	-	-	+	-	+	11	c.3710 C>T	p.A1237V	MS
25	69	III	-	-	-	+	+	+	+	11	c.1918 C>G	p.Q640E	MS
26	52	III	-	+	-	-	-	-	-	11	c.2212 G>A	p.V738I	MS
27	57	II	-	+	+	-	-	+	+	11	c.2312 T>C	p.L771S	MS
28	55	I	-	+	+	-	+	+	+	11	c.2363 T>C	p.V788A	MS
29	50	III	-	+	+	-	+	+	+	11	c.1492 C>G	p.L498V	MS
30	55	II	+	-	-	+	-	+	+	11	c.2741 A>G	p.E914G	MS
31	75	II	-	-	-	+	-	+	+	11	c.1791 A>T	p.E597D	MS
32	57	II	+	-	+	+	+	+	+	11	c.2741 A>G	p.E914G	MS
33	49	I	-	-	+	-	-	+	+	2	c.61 A>G	p.I21V	MS
34	68	II	-	+	+	-	-	+	+	11	c.1010delA	p.K339RfsX2	FS
35	61	II	-	+	+	+	-	-	+	3	c.123 C>G	p.H41Q	MS
36	77	I	-	+	+	-	-	-	-	11	c.4046_4047insG	p.E1352GfsX4	FS
37	53	II	+	-	-	+	+	+	+	14	c.4372 C>T	p.Q1458X	NS
38	80	II	-	+	+	-	-	+	+	11	c.1974 G>C	p.M658I	MS

39	80	I	+	+	-	+	-	+	+	24	c.5666 G>C	p.G1889A	MS
40	62	II	-	+	+	+	-	+	+	11	c.2138-2139delCA	p.S713X	NS
41	87	II	-	+	+	-	-	+	+	11	c.2408 G>C	p.S803T	MS
42	48	I	-	+	+	-	+	-	+	24	c.6677 C>G	p.S2226X	NS
43	47	II	+	+	+	-	-	+	+	11	c.2521 C>G	p.R841G	MS
44	57	II	-	+	+	+	-	+	+	11	c.2212_2213insC	p.V738AfsX1	FS
45	49	II	-	+	+	-	-	+	+	11	c.2954 C>G	p.P985R	MS
46	49	II	-	+	+	-	-	+	+	11	c.3290 G>C	p.S1097T	MS
47	56	I	+	+	+	-	-	-	+	11	c.3620-3621delAG	p.K1208IfsX11	FS
48	28	II	+	+	+	+	+	+	+	11	c.3827 T>A	p.L1276X	NS
49	73	II	-	+	+	-	-	-	+	11	c.3816 C>G	p.N1272K	MS
50	57	I	+	-	-	+	+	+	+	11	c.4135 T>A	p.S1379T	MS
51	59	II	-	-	+	-	-	-	+	12	c.4201 C>G	p.Q1401E	MS
52	46	II	-	-	-	+	+	-	-	12	c.4257 A>T	p.E1419D	MS
53	61	I	-	+	+	-	-	-	+	12	c.4297-4298delAT	p.I1433KfsX2	FS
54	49	II	+	+	+	+	-	+	+	11	c.2954 C>G	p.P985R	MS
55	55	II	+	+	+	-	-	-	+	7	c.349 C>G	p.H117D	MS
56	41	I	+	+	+	+	+	+	+	12	c.4200-4201delGC	p.M1400IfsX3	FS
57	79	II	-	+	-	-	-	+	+	13	c.4404 T>A	p.N1468K	MS
58	49	II	-	+	+	-	-	-	-	14	c.4578 G>C	p.E1526D	MS

59	56	I	-	-	-	+	+	-	+	14	c.4656 C>G	p.Y1552X	NS
60	56	III	-	+	+	+	-	+	+	15	c.4820 T>A	p.V1607D	MS
61	45	III	-	+	+	+	-	+	+	22	c.5379 G>C	p.K1793N	MS
62	50	II	-	-	-	-	-	+	-	20	c.5255 C>G	p.A1752G	MS
63	67	III	-	-	-	-	-	+	-	22	c.5390 C>A	p.S1797X	NS
64	58	III	+	+	+	-	-	+	+	24	c.5588 A>T	p.Y1863F	MS
65	71	II	+	+	+	-	-	+	+	24	c.5671 C>G	p.P1891A	MS
66	38	I	+	-	-	+	-	+	+	24	c.5588 A>T	p.Y1863F	MS
67	52	III	+	-	-	-	+	+	-	24	c.6324_6325insG	p.C2109VfsX1	FS
68	56	I	-	+	+	+	-	+	+	24	c.5883 C>G	p.F1961L	MS

a, NS: nonsense mutation; MS: missense mutation; FS: Frameshift mutation

Supplementary Table 3. List of commercial antibodies and lentiviral particles

Antibody	Company (catalog number)	Description
H3K9Ac	Abcam (ab4441)	ChIP
H3K18Ac	Abcam (ab1191)	ChIP
H3K27Ac	Millipore (07-360)	ChIP
H3K4me1	Abcam (ab8895)	ChIP
H3K4me2	Abcam (ab7766)	ChIP
H3K4me3	Abcam (ab8580)	ChIP
H3K36me3	Abcam (ab9050)	ChIP
H3K79me	Abcam (ab2886)	ChIP
H3K9me	Abcam (ab9045)	ChIP
H3K9me2	Millipore (07-441)	ChIP
H3K9me3	Abcam (ab8898)	ChIP
H3K27me	Millipore (07-448)	ChIP
H3K27me2	Millipore (07-452)	ChIP
H3K27me3	Millipore (07-449)	ChIP
PEMT	Santa Cruz (sc-130842)	WB, IHC
GCN5	Santa Cruz (sc-37946-V)	shRNAs
PCAF	Santa Cruz (sc-36198-V)	shRNAs
HLCS	Santa Cruz (sc-72198-V)	shRNAs
EHMT1	Santa Cruz (sc-62261-V)	shRNAs
GCN5	Santa Cruz (sc-20698)	WB
PCAF	Santa Cruz (sc-13124)	WB
HLCS	Santa Cruz (sc-23734)	WB
EHMT1	Santa Cruz (sc-130747)	WB

List of abbreviations used: ChIP, chromatin immunoprecipitation; shRNAs, short hairpin RNAs; WB, western blotting; IHC, immunohistochemistry.

Supplementary Table 4. Primers used in this study

Gene	Primers	Description
PEMT1-BSP-F	5’- GAATGAATGAAGGAAAAAATGAG	Methylation analysis for PEMT
PEMT1-BSP-R	5’- TCAATAAAACACAATCCCAAAAA	promoter 1 (Round I)
PEMT1-BSP-F	5’- TGAATGAAGGAAAAAATGAG	Methylation analysis for PEMT
PEMT1-BSP-R	5’- CCCCATTACCTAAAAACTAA	promoter 1 (Round II)
PEMT2-BSP-F	5’- TTTTATGTGGAAAAATAGGG	Methylation analysis for PEMT
PEMT2-BSP-R	5’- ACACATAAACAAACAACAAAC	promoter 2 (Round I)
PEMT2-BSP-F	5’- TTATTTTTGTTGTTTAGGTTGGAGTG	Methylation analysis for PEMT
PEMT2-BSP-R	5’- ACAAATCCTCCTAAAATCCATCAC	promoter 2 (Round II)
PEMT3-BSP-F	5’- GTAGAGATAGGGTTTTATTA	Methylation analysis for PEMT
PEMT3-BSP-R	5’- CCAAATAAAAAAATCAACACCTC	promoter 3 (Round I)
PEMT3-BSP-F	5’- GAGGGAGATTTTGTTTTAAA	Methylation analysis for PEMT
PEMT3-BSP-R	5’- AACAATAATAATATACCTCTCCTC	promoter 3 (Round II)
PEMT1-MSP-MF	5’- GTTTTTAGTTTTATTGTTGTTAGGTAA	Methylation analysis for PEMT
PEMT1-MSP-MR	5’- AAATACCTCTTTATCTAAAAACTCACG	promoter 1
PEMT1-MSP-UF	5’- GTTTTTAGTTTTATTGTTGTTAGGTAA	Methylation analysis for PEMT
PEMT1-MSP-UR	5’- AAATACCTCTTTATCTAAAAACTCACA	promoter 1
PEMT-total-RTP-F	5’- TCAAGGAGGCGAGAGTGA	Real-time PCR for transcript 1, 2
PEMT-total-RTP-R	5’- CCAGGTAGTTGGCTGTGCTT	and 3
PEMT1-RTP-F	5’- GGATGAAGAGATCTGGGAAC	Real-time PCR for transcript 1
PEMT1-RTP-R	5’- TGAAGGTGATGGTGATGACG	
PEMT2-RTP-F	5’- TGAGGCTATTCGTCGGTGAC	Real-time PCR for transcript 2
PEMT2-RTP-R	5’- TGAAGGTGATGGTGATGACG	
PEMT3-RTP-F	5’- TTGCTTTTACCTGATTCCTG	Real-time PCR for transcript 3
PEMT3-RTP-R	5’- GATTGAAGGTGATGGTGATG	

GAPDH-RTP-F	5'- AGGTGAAGGTCGGAGTCA	Real-time PCR for GAPDH
GAPDH-RTP-R	5'- GGTCATTGATGGCAACAA	
GCN5-SQP	Santa Cruz Biotech GCN5 (h)-PR: sc-37946-PR	Semi-quantitative PCR for GCN5
PCAF-SQP	Santa Cruz Biotech PCAF (h)-PR: sc-36198-PR	Semi-quantitative PCR for PCAF
HLCS-SQP	Santa Cruz Biotech HLCS (h)-PR: sc-72198-PR	Semi-quantitative PCR for HLCS
EHMT1-SQP	Santa Cruz Biotech EHMT1 (h)-PR: sc-62261-PR	Semi-quantitative PCR for EHMT1
PEMT1-ChIP-RTP-F	5'- TCCAAGGGGATACTGCTAC	ChIP for PEMT promoter 1
PEMT1-ChIP-RTP-R	5'- CAACATATTCCGGCCTTCT	
PEMT1-ChIP-SQP-F	5'- GATCCAAGGGGATACTGCTA	ChIP for PEMT promoter 1
PEMT1-ChIP-SQP-R	5'- TTCTGGGAAATGTAGTTCCC	
PEMT1-Mutation-F	5'-CTCCTCTAGACGCTGTTGCGAGCCCCC AGATAA	A point mutation at the site of -132 (C to T) in the first promoter of PEMT
PEMT1-Mutation-R	5'-TTATCTGGGGGCTCGCAACAGCGTCTA GAGGAG	

List of abbreviations used: BSP, bisulfite-sequencing PCR; MSP, methylation-specific PCR; RTP, real-time PCR; SQP, semi-quantitative PCR; ChIP, chromatin immunoprecipitation; F, forward primer; R, reverse primer; M, methylated; U, unmethylated.
