# Supplementary material for: Epigenetic repression of phosphatidylethanolamine N-methyltransferase (PEMT) in BRCA1-mutated breast cancer
Source: Oncotarget. 2014 Feb 27;5(5):1315–25. doi: 10.18632/oncotarget.1800 (PMC4012741; doi:10.18632/oncotarget.1800)
Supplement: Supplementary file 3 [file oncotarget-05-1315-s003.pdf]

Supplementary Methods

Bisulfite sequencing for differential PEMT promoter. Genomic DNA from breast cancer and adjacent normal breast tissues was extracted using a TIANamp Genomic DNA kit (Tiangen Biotech, Beijing, China), and was subjected to bisulfite conversion using the EZ DNA Methylation-Direct kit (Zymo Research, Orange, USA) following the manufacturer's instructions: the conversion efficiency was estimated to be at least 99.6%. It was then amplified by nested PCR. After gel purification, cloning and transformation into *E. coli* Competent Cells JM109 (TaKaRa, Dalian, China), ten positive clones of each sample were sequenced to ascertain the methylation patterns of each CpG locus. The specific primer sequences for bisulfite sequencing are described in Supplementary Table 3. The conditions were as follows: 95°C for 2 min; 40 cycles of 30 s at 95°C, 30 s at 56°C and 45 s at 72°C; then 72°C for 7 min.

Methylation-specific PCR for PEMT promoter 1. Genomic DNA extraction and sodium bisulfite conversion were performed according to the above procedure. The specific primer sequences for methylation-specific PCR are described in Supplementary Table 3. The conditions were as follows: 95°C for 5 min; 10 cycles of 30 s at 95°C, 45 s at 58°C and 45 s at 72°C; then 33 cycles of 30 s at 95°C, 45 s at 55°C and 45 s at 72°C; followed by 72°C for 10 min. To exclude false-positive and false-negative results, universal unmethylated DNA and universal methylated DNA were purchased from Chemicon (Temecula, CA, USA) and served as controls.

Genomic DNA methylation assay. The genomic DNA methylation was measured as described (1). Briefly, genomic DNA from the breast samples was extracted using a

TIANamp Genomic DNA kit (Tiangen Biotech). Contaminating RNA was removed by incubation with RNase A (100 μg/mL) and RNAse T1 (2000 unit/mL) for 2 hours at 37°C. Following the incubation, DNA was phenol/chloroform/isoamyl alcohol extracted, ethanol precipitated, resuspended in 100 μL DNase I digestion buffer (10 mM Tris-HCl, pH 7.2, 0.1 mM EDTA, 4 mM $MgCl_2$) and digested using DNase I (50 μg/mL) for 14 hours at 37°C. DNA was further digested using Nuclease P1 (50 μg/mL) for 7 hours at 37°C in the presence of 2 volumes of 30 mM sodium acetate (pH 5.2) and 1 mM zinc sulphate. Solid debris was removed by centrifugation using a spin column with a 0.45-μm filter. Hydrolysed DNA was analysed for cytosine methylation content by HPLC using a Hypersil ODS C18 column (Thermo, Bellefonte, PA, USA) with a mobile phase consisting of 50 mM ammonium orthophosphate (pH 4.1) at a flow rate of 1 mL/min and UV detection was performed at 278 nm. The amount of DNA cytosine methylation was calculated by the methylated cytosines/total cytosines in DNA×100 (%). All reagents were purchased from Sigma (CA, USA).

Real-time quantitative PCR. Total RNA was extracted using Trizol reagents (Invitrogen, CA USA) according to the manufacturer's protocol. DNA contamination was removed by adding DNase I (Invitrogen). Total RNA was then reverse-transcribed from 2 μg of RNA using the PrimeScript RT Master Mix kit (TaKaRa) and amplified by SYBR Premix Ex TaqTM II (TaKaRa) in a Roche LightCycler 2.0 instrument (Roche Diagnostics, Mannheim, Germany). The specific primer sequences for real-time PCR are listed in Supplementary Table 3. GAPDH mRNA was amplified as an internal control for the normalization of each sample. All samples were analyzed in triplicate using the $2^{-\Delta\Delta CT}$ method.

Immunohistochemistry. The standard SP kit (Zhongshan, Beijing, China) was used for

immunohistochemical staining. Briefly, serial 4 μm sections were obtained from each paraffin-embedded tissue block. Following deparaffinization and rehydration, sections were subjected to microwave antigen retrieval. The primary antibody was rabbit polyclonal anti-PEMT (1:100; Santa Cruz), and the sections were incubated overnight at 4°C with this antibody. 3,3'-diaminobenzidine was used as the chromogen. Nuclei were counterstained with hematoxylin, and slides were dried and mounted. Negative controls were incubated with phosphate-buffered saline instead of the antibody. Immunostaining was evaluated by two independent pathologists, blinded to the identity of the subject groups. Area quantification was made with a light microscope at a magnification of 400 × and analyzed by Image-Pro Plus 6.0 (Media 2 Cybernetics, USA) using PEMT-positive cells.

Western blotting. Knockdown efficiency of GCN5, PCAF, HLCS and EHMT-1 was assayed by western blotting. Western blotting analysis was performed 48 h after transfection according to standard protocols. Briefly, protein concentration was determined by the Bio-Rad Protein Assay Kit (Hercules, CA, USA). 30 μg of protein was separated by 8% SDS polyacrylamide gels, and transferred to polyvinyl difluoride membranes (Millipore, MA, USA). The membranes were blocked in TBS containing 0.1% Tween-20 and 5% non-fat dry milk for 60 min at room temperature, and incubated with antibody overnight at 4°C (see details of antibody information in Supplementary Table 2). Then, the membranes were washed by PBS-Tween followed by 1 h incubation at room temperature with horseradish peroxidase-conjugated secondary antibody (1:5000; Santa Cruz) and detected using enhanced chemiluminescence (Amersham Life Science, NJ, USA).

Chromatin immunoprecipitation assay (ChIP). ChIP assays were performed using the EpiQuik™ Tissue Chromatin Immunoprecipitation kit (Epigentek Group Inc., Brooklyn, NY,

USA) according to the manufacturer's instructions. Briefly, the cultured cells and small pieces of breast tissues (1–2 mm^3) were crosslinked with 1% formaldehyde. Cross-linking was terminated using 1.25 M glycine. The tissues and cells were added to homogenizing buffer, triturated, disaggregated, and centrifuged at 1000 g for 5 min at 4°C. After the removal of supernatants, the protease inhibitors were added and the disaggregated tissue pellet was resuspended. Chromatin was sheared by sonication. Immunoprecipitation was performed at room temperature for 90 min. The specific antibodies for ChIP are provided in Supplementary Table 2. Crosslinking was reversed at 65°C for 90 min. Eventually, genomic DNA was eluted for PCR analysis. The specific primer sequences for ChIP are provided in Supplementary Table 3. The thermocycle was 94°C for 2 min, then 30 cycles of 45 s at 94°C, 45 s at 56°C and 45 s at 72°C.

Generation of PEMT promoter 1 luciferase constructs with -132 site-directed mutagenesis or methylation. Genomic DNA from breast tissues was extracted using a TIANamp Genomic DNA kit (Tiangen Biotech). PEMT promoter 1 was obtained by PCR amplification. The specific primer sequences are provided in Supplementary Table 3. The fragments were sub-cloned into the luciferase reporter plasmid pGL4 enhancer vector (Promega, Madison, USA). For site-directed mutagenesis, the luciferase reporter plasmid was used as a template to insert a C to T base mutation (-132 site) following the manufacturer's protocol (Stratagene, La Jolla, CA, USA). The specific primer sequences for site-directed mutagenesis are provided in Supplementary Table 3. Methylated -132 site DNA was synthesized by Sangon Biotech Ltd (Shanghai, China).

Transfection and dual-luciferase reporter assay. The 293T cells, and primary non-mutated and BRCA1-mutated breast cancer and their normal breast cells were plated onto 24-well

plates. All transfections were performed using Lipofectamine™ 2000 (Invitrogen) according to the manufacturer's protocol. For reporter gene experiments, the different desired PEMT pGL4-Enhance constructs (0.8 μg per well) were co-transfected with the reference renilla luciferase reporter plasmid (pRL-TK, 0.1 μg per well) to normalize for transfection efficiency. Following 24 h of culture, cell lysates were prepared according to the recommendations of the Dual Luciferase Reporter Assay System® manufacturer (Promega). Then, firefly and renilla luciferase products were measured. Firefly luciferase activity was normalized to renilla luciferase activity and expressed as fold induction. The fold induction represents the transcriptional activity driven by a particular luciferase reporter construct.

References

1. Ramsahoye BH. Measurement of genome wide DNA methylation by reversed-phase high-performance liquid chromatography. Methods 2002;27:156-61.
